# Electrical Characterization of Amorphous Silicon MIS-Based Structures for HIT Solar Cell Applications

**DOI:** 10.1186/s11671-016-1545-z

**Published:** 2016-07-16

**Authors:** Héctor García, Helena Castán, Salvador Dueñas, Luis Bailón, Rodrigo García-Hernansanz, Javier Olea, Álvaro del Prado, Ignacio Mártil

**Affiliations:** Department of Electronics, University of Valladolid, Paseo Belén, 15, 47011 Valladolid, Spain; Dpto. Física Aplicada III, Universidad Complutense de Madrid, CEI Campus Moncloa, UCM-UPM, Madrid, Spain

**Keywords:** Hydrogenated amorphous silicon, Photovoltaics, Interfacial state density, Deposition temperature dependencies, Electron cyclotron resonance chemical vapor deposition

## Abstract

A complete electrical characterization of hydrogenated amorphous silicon layers (a-Si:H) deposited on crystalline silicon (c-Si) substrates by electron cyclotron resonance chemical vapor deposition (ECR-CVD) was carried out. These structures are of interest for photovoltaic applications. Different growth temperatures between 30 and 200 °C were used. A rapid thermal annealing in forming gas atmosphere at 200 °C during 10 min was applied after the metallization process. The evolution of interfacial state density with the deposition temperature indicates a better interface passivation at higher growth temperatures. However, in these cases, an important contribution of slow states is detected as well. Thus, using intermediate growth temperatures (100–150 °C) might be the best choice.

## Background

Amorphous silicon layers are of particular interest for photovoltaic applications [1]. In fact, as thin amorphous silicon (a-Si) layers saturate the crystalline silicon (c-Si) surfaces, the formation of recombination centers is avoided, and high quality interfaces are formed, which is of great interest for heterojunction with intrinsic thin layer (HIT) cells [2]. This kind of solar cell consists of a crystalline/amorphous silicon heterojunction, and between both layers, a very thin film (~5 nm) of intrinsic amorphous silicon (i-a-Si:H) is introduced. The i-a-Si:H layer saturates the silicon surface dangling bonds, and therefore, this surface is passivated. Based on this technology, Panasonic achieved the efficiency world record with 25.6 % for silicon-based solar cells [3]. Plasma-enhanced chemical vapor deposition (PECVD) is the most extended technique used to deposit a-Si:H [4, 5]. In this technique, the substrate and the plasma are very close, thus the substrate surface could be damaged by plasma bombardment. In this work, we used the electron cyclotron resonance chemical vapor deposition (ECR-CVD) technique to deposit the a-Si:H, which is a remote plasma procedure [6] that could reduce the damage to the c-Si surface. Other advantages of ECR-CVD are the possibility of in situ substrate plasma pre-treatment [7] and low processing pressure, which permits minimizing the contamination of the growing film [8]. The possibility for scaling-up makes the ECR-CVD a very attractive technique for commercial solar cell fabrication.

As the aim of the intrinsic a-Si:H is the c-Si surface passivation, an interface study in depth is desirable. Previous works showed that the intrinsic amorphous silicon behaves as an insulator at low bias [9], and therefore, it is possible to apply the metal-insulator-semiconductor (MIS) characterization techniques in order to extract information from the a-Si:H/c-Si heterointerface. The density of interface defects can be related to the silicon surface dangling bonds, and its analysis is desirable in order to improve the a-Si:H quality [10].

In this work, we present a characterization of the interface between a-Si:H and c-Si. The results presented in this work support the use of a-Si:H thin films in HIT solar cells.

## Methods

Metal—intrinsic amorphous silicon—semiconductor structures were fabricated as follows: 100 nm of intrinsic a-Si:H was deposited by ECR-CVD with an Astex-4500 reactor on n-type <100> silicon wafers with a resistivity of 1–10 Ωcm. Before deposition, the substrates were cleaned by a standard Radio Corporation America (RCA) cleaning [11] process, followed by a dip in diluted HF. The microwave power supply generator was 100 W. The precursor gas used was 19 sccm of 95 % Ar + 5 % SiH_4_. Four different deposition temperatures were used (30, 100, 150, and 200 °C). Metal electrodes (100 nm Ti + 200 nm Al) were deposited by e-beam evaporation. An annealing treatment was performed in forming gas atmosphere at 200 °C during 10 min by using RTP-600S equipment from Modular Process Technology Corp. The area of measured devices was 1.7 × 10^−4^ cm^2^. Fourier transform infrared spectroscopy (FTIR) measurements were performed by using a Nicolet Magna-IR 752 spectrometer, in a wave number range from 340 to 4000 cm^−1^.

As it was said before, due to its low carrier density, a-Si layers behave like insulator layers, so fabricated structures exhibit a similar behavior to MIS capacitors. Therefore, their study was carried out by using the electrical characterization techniques developed for MIS structures. Electrical measurements were carried out putting the sample in a light-tight, electrically shielded box. In order to record electrical parameters at temperatures from liquid nitrogen temperature (≈77 K), samples were cooled in an Oxford DM1710 cryostat. An Oxford ITC 502 temperature controller was used to keep the temperature constant while the electrical measurements are carried out. Current-voltage (*I-V*) curves were measured using the HP-4155B semiconductor parameter analyzer. Capacitance-voltage (*C*-*V*) and conductance-voltage (*G*-*V*) measurement setups involved a Keithley 4200SCS semiconductor analyzer. The experimental setup of the conductance transient technique consisted of an HP 3310A function generator to apply the bias pulses, an EG&G 5206 two-phase lock-in analyzer to measure the conductance, and an HP 54501A digital oscilloscope to record the complete conductance transients. Interface trap density (*D*_it_) was measured by deep-level transient spectroscopy (DLTS). These measurements were performed using a Boonton 72B capacitance meter, an HP 54501A digital oscilloscope to record the capacitance transients, and an HP 8112A pulse generator to bias the samples from inversion to accumulation. Finally, to obtain the flat-band voltage (*V*_FB_) transients, a feedback system varied the applied gate voltage accordingly to keep the capacitance at its flat-band value. An Agilent N6700B bias source, a Keithley 6517A electrometer, and a Boonton 72B capacitance meter were used for recording *V*_FB_ transients.

## Results and Discussion

**Fig. 1:**
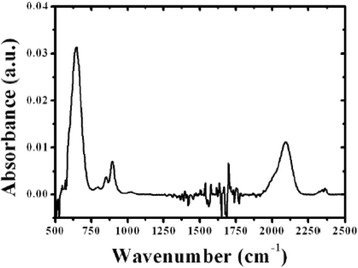
FTIR spectrum for one sample deposited by ECR-CVD at room temperature. All the bands are related to Si-H bonds

**Fig. 2:**
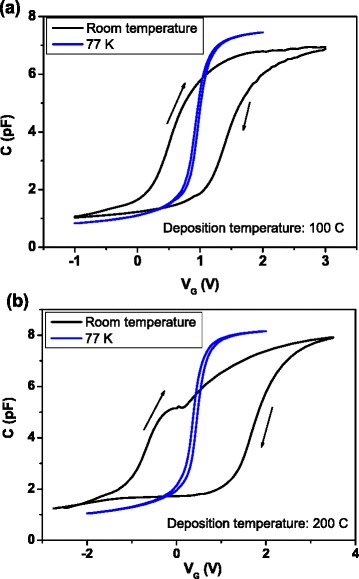
Capacitance-voltage curves measured at room temperature and at 77 K corresponding to a temperature deposition of 100 °C (**a**) and 200 °C (**b**)

**Fig. 3:**
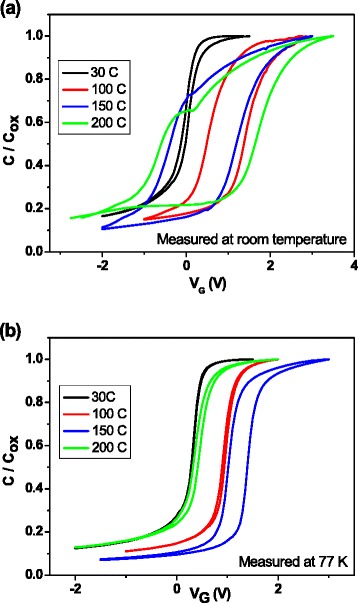
Capacitance-voltage curves obtained for all deposition temperatures, measured at room temperature (**a**) and at 77 K (**b**)

In Fig. 1, the characteristic FTIR spectrum of samples deposited at room temperature is shown. Two bands are observed at 640 and 2090 cm^−1^, corresponding to the Si-H wagging mode [12] and the Si-H_2_ stretching mode, respectively, as well as the Si-H_2_ bending mode in 850 cm^−1^ and the Si-H_2_ scissor mode in 894 cm^−1^ [13]. The presence of only Si-H-related bonds reveals high purity film composition. Figure 2a shows *C-V* measured at room and low temperatures for a sample grown at 100 °C. At room temperature, there is a clear hysteresis, which nearly vanishes at 77 K, thus indicating that the border traps, i.e., the defects located into the insulator in the region close to the interface insulator/oxide, are thermally activated. The same curves are shown in Fig. 2b for a sample grown at 200 °C. In this case, hysteresis at room temperature is even greater than that observed in the sample grown at 100 °C. Also, a “shoulder”-shaped feature is clearly seen. Samples grown at the lowest temperatures do not exhibit such feature (see Fig. 3a), so this seems to appear only in samples grown at the highest temperatures, and it is similar to the kink in the *C-V* response reported in other works [14, 15]. As growth temperature increases, hysteresis effect and stretch-out of *C-V* curves increase as well. *C-V* curves measured at 77 K (Fig. 3b) show much lower hysteresis than those obtained at room temperature, thus indicating that some traps in the material are frozen at low temperatures [16]. Sample grown at 150 °C still exhibits appreciable hysteresis at 77 K. As for flat-band voltage values, they increase with growth temperature, reach the maximum value at 150 °C, and then return to the lowest value, as it is shown in Fig. 3b.

**Fig. 4:**
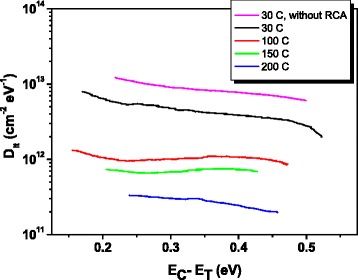
Interfacial state densities measured by DLTS corresponding to all deposition temperatures. *D*_it_ profile obtained for a sample grown at 30 °C without previous RCA treatment is also shown for comparison

Interfacial state densities measured by DLTS are shown in Fig. 4. This technique is time sensitive, so contributions with different time constants are separated and the fast contributions of interface states are clearly distinguished from the slow ones corresponding to defects in the oxide bulk. To obtain the interfacial trap distribution within the band gap, the MIS capacitor is biased just at the limit between depletion and weak inversion, and a pulse high enough to drive the capacitors into the accumulation regime is then applied in order to fill all interfacial traps. The use of a digital oscilloscope allows recording the entire capacitance transients, and therefore, the entire energy spectrum is processed with only one temperature scan. The lowest value of *D*_it_ (2–3 × 10^11^ cm^−2^ eV^−1^) corresponds to the sample grown at 200 °C. For intermediate growth temperatures, 100 and 150 °C, *D*_it_ values are around 7 × 10^11^–1 × 10^12^ cm^−2^ eV^−1^. These values can be considered acceptable. When growth temperature is 30 °C, *D*_it_ increases until it reaches values near 1 × 10^13^ cm^−2^ eV^−1^. This value is too high for technological applications. For comparison, Fig. 4 also depicts the *D*_it_ profile corresponding to a sample grown at 30 °C without a previous cleaning stage by RCA. In this case, *D*_it_ values are even higher.

**Fig. 5:**
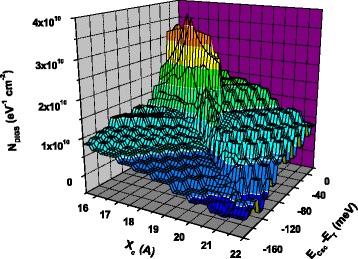
Border trap three-dimensional distribution of a sample grown at 100 °C

Border trap densities obtained by means of conductance transient analysis are of the same order of magnitude for the samples grown at 100, 150, and 200 °C. As an illustration, Fig. 5 shows the three-dimensional plot of disorder-induced gap state density (*N*_DIGS_) as a function of the energy and the spatial coordinate for the sample grown at 100 °C. It can be seen that *N*_DIGS_ values are in the range of standard MIS devices. On the other hand, for the sample grown at 30 °C, conductance transient amplitudes are below the experimental resolution, so we can conclude that its border trap density is lower than 1 × 10^10^ cm^−2^ eV^−1^. This result agrees well with the high value of *D*_it_ measured in this sample, because in general, when border trap density (*N*_DIGS_) is high, interfacial state density (*D*_it_) is low [17]. So, for the sample grown at 30 °C, fast traps are preferentially located at the insulator/semiconductor interface, whereas for samples grown at 100–200 °C, fast traps are defects located both at the interface and in the insulator.

**Fig. 6:**
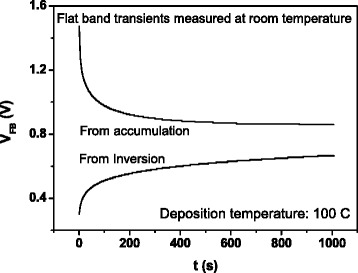
Flat-band transients measured at room temperature from accumulation and from inversion, corresponding to a sample grown at 100 °C

**Fig. 7:**
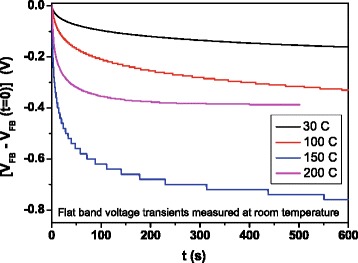
Flat-band transients from accumulation measured at room temperature corresponding to all deposition temperatures

**Fig. 8:**
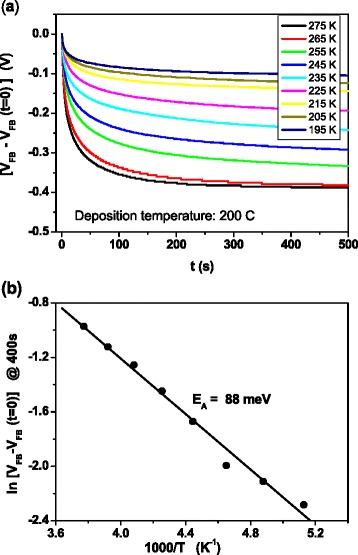
Flat-band transients from accumulation measured at different temperatures (**a**) and Arrhenius plot (**b**) of a sample grown at 200 °C

Figures 6, 7, and 8 are related to flat-band transient recordings. Figure 6 demonstrates for the sample grown at 100 °C that there are flat-band transients at room temperature when the sample is previously biased in accumulation or in inversion for a few seconds. In both cases, it is possible to obtain an Arrhenius plot by recording transients at different measurement temperatures. Measurements were carried out at a voltage bias of 0 V, keeping the capacitance at its flat-band value. The flat-band voltage transients are due to trap charging and discharging of defects inside the oxide layer. This mechanism gives rise to variations in flat-band voltage value due to changes in the oxide charge density [18]. As the charge trapping or detrapping inside the oxide is usually tunneling assisted, states located deeper in the oxide capture carriers after those located near the semiconductor/oxide interface, so the *V*_FB_ transients obtained are expected to be slower than the DLTS transients. In Fig. 7, we can see that the transient amplitudes initially increase with growth temperature, reach their maximum value at 150 °C, and then diminish while transients become faster. In Fig. 8a, we show the flat-band voltage transients recorded at temperatures varying between 195 and 275 K for the sample grown at 200 °C. The amplitude of transients is thermally enhanced, and the temperature dependence of the transient amplitude follows an Arrhenius plot (Fig. 8b). The linear fit provides an activation energy value of 88 meV. Similar values are obtained for all the samples. In the case of high-k dielectrics, the activation energy has been found to be similar to the soft optical phonon energies [19].

**Fig. 9:**
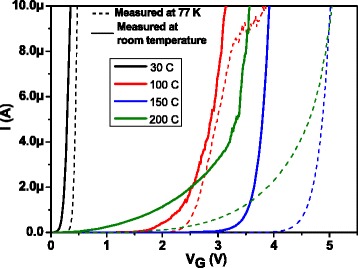
Current-voltage curves measured at room temperature and 77 K corresponding to all deposition temperatures

Figure 9 shows current-voltage curves corresponding to the accumulation regime, measured at room temperature and at 77 K. The best behavior corresponds to the 150 °C-deposited sample, with breakdown electric field values corresponding to 2 mA/cm^2^ of around 0.35 and 0.45 MV/cm, at room temperature and 77 K, respectively.

**Fig. 10:**
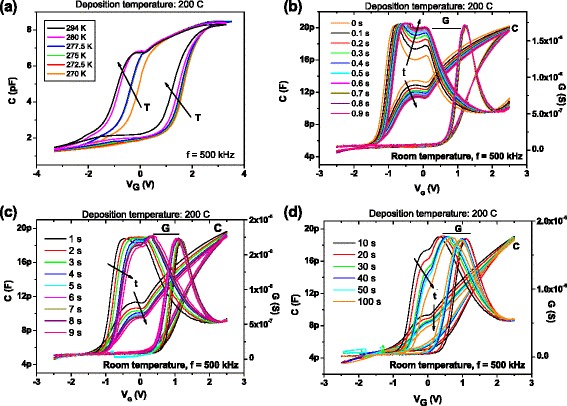
Capacitance and conductance vs. bias hysteresis loops measured at 500 kHz corresponding to a sample grown at 200 °C, at different temperatures, by using a voltage ramp (**a**), and at room temperature, by using a stair-shaped voltage, with different values of the step width (**b**–**d**). In all cases, the step of voltage bias was 20 mV

Finally, to study in depth the “shoulder”-shaped feature showed in Fig. 2b, some additional measurements have been carried out. When measurement temperature diminishes, the feature also diminishes (see Fig. 10a). In fact, at temperatures lower than 270–280 K, it completely disappears. Focusing on the *C-V* and *G-V* curves at room temperature, in the next experiment (Fig. 10b–d), a stair-shaped voltage was used instead of a voltage ramp, with different values of the step width, from 0 s (which corresponds to the voltage ramp) until 100 s. The absolute value of the increment of the voltage bias was 20 mV in all measurements. Both capacitance and conductance signals exhibit a very different behavior if voltage goes from accumulation to weak inversion or vice versa. In the former case, *C-V* and *G-V* curves indicate that generation and recombination through interface trap levels dominate the loss. On the contrary, when voltage bias passes from negative to positive values, *C-V* curves show a kink and *G-V* curves show two peaks instead of only one. One of these peaks remains nearly in the same voltage position when measurement conditions are changed. The second one appears at lower values of the voltage and clearly moves to higher values when the measurement time increases. Also, this peak diminishes and nearly disappears for time values of around 100 s.

The shape of *C-V* and *G-V* curves when voltage bias values increase can be attributed to the discharge process of slow states located far from the interface, which need high temperature or longer time to release their positive charge. When charge emission takes place, flat-band voltage moves towards more positive values, and so the hysteresis width of *C-V* curves diminishes. At the same time, the shoulder-shaped feature vanishes. As for *G-V* curves, the second peak moves to the right and its height diminishes. So, when the samples are driven from weak inversion to accumulation, the loss is due to the generation and recombination through both bulk and interfacial trap levels.

As indicated in Fig. 3a, these phenomena do not appear in samples grown at low temperatures, so the presence of slow traps in the a-Si layer bulk seems to be related to the growth processes carried out at temperatures above 150 °C.

## Conclusions

Interfacial state density values for samples grown at temperatures of 100 °C and above are in the range of standard devices. Border trap densities and activation energy values are in the standard values as well. From our experimental results, the optimal amorphous/crystalline silicon interface is achieved for the highest growth temperature value. However, in samples grown at temperatures of above 150 °C, a significant presence of slow traps in the a-Si layer is detected. In conclusion, to use these kinds of structures in the HIT solar cell application field, growing a-Si layers at 100–150 °C seems to be the most adequate choice.

## Abbreviations

a-Si:H, hydrogenated amorphous silicon layers; c-Si, crystalline silicon; *C-V*, capacitance-voltage; *D*_it_, interface trap density; DLTS, deep-level transient spectroscopy; ECR-CVD, electron cyclotron resonance chemical vapor deposition; FTIR, Fourier transform infrared spectroscopy; *G-V*, conductance-voltage; HIT, heterojunction with intrinsic thin layer; i-a-Si:H, intrinsic amorphous silicon; *I-V*, current-voltage; MIS, metal-insulator-semiconductor; *N*_DIGS_, disorder-induced gap state density; PECVD, plasma-enhanced chemical vapor deposition; RCA, Radio Corporation America; *V*_FB_, flat-band voltage
